# Practices supporting cue-based breastfeeding of preterm infants in neonatal intensive care units across Europe

**DOI:** 10.1186/s13006-024-00697-y

**Published:** 2025-01-02

**Authors:** Bente Silnes Tandberg, Hege Grundt, Ragnhild Maastrup, Annie Aloysius, Livia Nagy, Renée Flacking

**Affiliations:** 1 Department of Paediatric and Adolescent Medicine, Drammen Hospital, Vestre Viken Hospital Trust, Vestre Viken HT, Post Box 800, Drammen, 3004 Norway; 2 Lovisenberg Diaconal University College, Oslo, Norway; 3 Department of Neonatology, Haukeland University Hospital, Bergen, Norway; 4 Knowledge Centre for Breastfeeding Infants with Special Needs, Department of Neonatology, Copenhagen University Hospital Rigshospitalet, Copenhagen, Denmark; 5 Department of Neonatology, Imperial College Healthcare NHS Trust, London, UK; 6 Melletted a helyem Egyesület (Right(s) Beside You Association), Budapest, Hungary; 7 School of Health and Welfare, Dalarna University, Falun, Sweden

**Keywords:** Cue-based feeding, Breastfeeding, Neonatal intensive care, Neonatal intensive care unit, Preterm infant

## Abstract

**Background:**

Emerging knowledge about supportive neurodevelopmental neonatal care shows the need for an individual approach to establish breastfeeding. However, evidence on how cue-based breastfeeding is supported in neonatal intensive care units (NICUs) is scarce. Therefore, the aim was to describe supporting practices for cue-based breastfeeding.

**Method:**

Through Delphi rounds, a questionnaire was developed comprising questions on the usage and occurrence of supportive practices for cue-based breastfeeding. A multinational online survey was distributed September to October in 2023 to NICUs in Europe using snowball sampling. Practices such as the practice of skin-to-skin contact (SSC), restrictions for breastfeeding, providing information to parents, observing and responding to infants’ cues were explored.

**Results:**

The survey was completed by 105 neonatal units across 15 European countries. Less than half (46%) of the NICUs had no restrictions upon placing the infant in SSC with the parents. Approximately half (49%) of the NICUs stated that infants had SSC within the first hour after birth. Many units (68%) had some restriction for breastfeeding. One week after birth, 48% of the NICUs encouraged breastfeeding for infants at 33 postmenstrual age whenever the infant showed cues, regardless of scheduled tube feeding time. This percentage increased to 59% at 33–35 gestational age. Less than half of the units (47%) stated that they had the necessary tools/instruments to support the transition from tube feeding to breastfeeding. There were variations in how milk intake was assessed, such as weighing before and after breastfeeding or estimating milk intake by time spent sucking. Infants in 50% of the units had to be fed exclusively orally before discharge. Many units (65%) provided specific support to or enabled discharge before the infant was exclusively orally fed.

**Conclusion:**

European NICUs employ supportive practices, SSC, early initiation of breastfeeding, and provide information to parents. Staff plays a significant role in fostering cue-based feeding in preterm infant-mother dyads. There still exist restrictions for SSC and breastfeeding. To understand the impact of different strategies and practices, there is need for evaluations by parents and testing of the implementation of cue-based feeding practices in neonatal care.

## Background

Breastfeeding a preterm infant is a complex process, often progressing slowly towards the establishment of exclusive breastfeeding [1, 2]. This progression is highly dependent on the infant’s gestational age at birth; infants of a younger gestational age have a more immature sucking skill, coordination of sucking-swallowing-breathing, and state regulation with fewer and shorter awake-alert periods [3, 4]. Evidence strongly recommends that preterm infants start with enteral and oral feeding of the mother’s milk on the first day of life or as soon as possible after birth [5]. For most preterm infants, the main enteral feeding method is by feeding tube.

Supporting an individual infant’s feeding journey is challenging for parents and professionals as preterm infants need time and support to develop feeding skills to grow and mature. Despite knowledge of the preterm infant’s capacities and development, restrictive policies on breastfeeding are common, such as on breastfeeding initiation, frequency of feeding times or duration of breastfeeding [6–9]. Mothers who have experienced such policies struggle to see positive responses in their infants during breastfeeding [10]. To date, most related research has focused on the nutritional aspects of breastfeeding, barriers for initiating breastfeeding and early weaning. Even so, in the last decade many professionals have advocated for providing a neurodevelopmentally supportive and dyadic approach for the establishment of breastfeeding [11, 12]. Numerous concepts in the literature describe this supportive feeding as cue-based feeding [11–16], responsive feeding [17], infant-driven feeding [18–20] and attuned feeding [21]. These are all euphemisms for approaches whereby the infant’s cues—such as behavioural responses and physiological signals—should guide the process towards exclusive breastfeeding or any oral feeding. The term *cue-based feeding* will be used hereafter, in which it is defined as observing and responding to infants’ feeding cues. In practice, this means that feeding starts when the infant shows cues that indicate readiness to feed. Readiness may include waking before scheduled tube feed times, rooting reflex, turning towards and looking at the breast, sucking their hands or fingers. The feeding should end when the infant demonstrates behavioural cues of disengagement, physiological instability, distress, satiety or falls asleep [12, 14]. Thus, the infant determines the timing, duration and volume of intake regardless of the feeding method. When enabling cue-based feeding, sufficient growth must also be prioritized. In a review, several studies showed favourable outcomes for cue-based bottle feeding compared to scheduled feeding [17]. To support cue-based breastfeeding while securing sufficient growth can be even more demanding due to the challenge of measuring the exact intake of volume of milk.

Studies on cue-based breastfeeding have mainly involved qualitative designs or quality improvement projects [11, 22]. However, recommendations suggest that an individualised and dyadic approach to the establishment of breastfeeding should be taken and that traditional scheduled feeding approaches, which prescribe volume and times, should not be recommended [23]. Several recommendations and standards [4, 5, 24] also state that a key practice in infant feeding is to support mothers in recognizing and responding to their infants’ feeding cues [24]. Subsequently, staff need to have the knowledge and skills required to guide and support mothers and infants. In this process, several tools are also available for staff to use in their support for mothers [4, 25–27]. The mother must be present to respond to the infant’s cues and to provide prolonged periods of skin-to-skin contact (SSC), both of which are important for breastfeeding per se and for cue-based feeding [28–30]. For many mother-infant dyads, the end of hospital stay is a period that involves an increased focus on nutrition and feeding, as the discharge criteria of being fed without a feeding tube is commonly used. To reduce stress and shorten the hospital stay, early discharge programs have been increasingly implemented [31, 32]. Such programs, in which preterm infants are discharged home while still on tube feeding and establishing oral feeding has been reported supportive for breastfeeding [33, 34].

In summary, support for cue-based breastfeeding involves practices such as SSC and enabling mothers to breastfeed based on the infant’s cues. Support also includes staff training, NICU guidelines and tools, and the transfer of knowledge to parents. Thus, arguments in favour of a neurodevelopmentally supportive approach have resulted in a shift towards cue-based feeding. However, there is a lack of knowledge as to what practices are used to support cue-based breastfeeding in NICUs. The aim was therefore to describe the occurrence of supporting practices for cue-based breastfeeding in NICUs across Europe.

## Methods

### Design

This study was conducted as a cross-sectional, multinational online survey.

### Development and content of the questionnaire

A questionnaire designed to describe how cue-based breastfeeding was supported and implemented in European NICUs was developed within a multidisciplinary, multinational network of clinicians, researchers and parents with patients’ expertise. This network, the Positive Feeding Neonatal Network, has broad and in-depth knowledge of, and experience with, feeding in preterm infants. Through discussions based on available evidence, the main practices relevant to support breastfeeding were identified: SSC, information and guidance to parents, observing and responding to infants’ cues, assessment of milk intake, support for staff through training, guidelines and tools, and support related to discharge.

The first version of the questionnaire was piloted in 28 European NICUs from six countries. The results of the pilot were presented at a workshop at the Separation and Closeness Experiences in the Neonatal Environment (SCENE) research network symposium in April 2023, and participants provided valuable feedback on the preliminary results and content of the questionnaire. Specifically, there was a need to re-word for better cultural adaptation and for adding more alternatives to multiple-choice questions. The final questionnaire comprised 31 multiple choice questions and one Likert scale related to current feeding practices and breastfeeding support. Questions were asked about the current practices in NICUs at set time points for a preterm infant: at birth, the first week, the intermediate phase during hospitalization, and at discharge. There were also questions related to practices to support cue-based breastfeeding in preterm infants, including SSC, information provided to parents, observing and responding to infants’ cues and assessment of milk intake, staff training, NICU guidelines and tools, and breastfeeding support related to discharge.

**Fig. 1:**
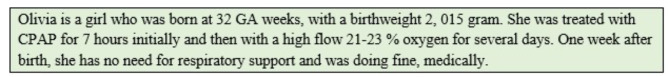
The case of Olivia. Legend: The case which the respondents were asked to refer to when answering the questionnaire

Because of the heterogeneous population in neonatal units, we chose to present the case of “Olivia” to help focus answers on a “typical” yet potentially complex case (Fig. 1).

The questionnaire included some overarching questions, such as “How are parents taught/informed about baby’s feeding cues?” as well as questions related to the presented case, “Olivia”, such as, “When her mother is present, when would Olivia be breastfed during weeks 33–35?”

In the questionnaire, breastfeeding was defined as feeding at the breast, and cue-based feeding as “observing and responding to babies’ cues”. In the introduction to the survey, we emphasized that the NICUs should respond to how they usually did something, not how units intended to do it or would ideally like to do it. The respondents had several statements to choose from and had the option of adding additional information or elaborating on practices. Where appropriate, it was possible to choose more than one answer. The characteristics of participating NICUs, such as nationality, name of the hospital and authorized/designated number of patients in the unit, were collected for description and validation, ensuring only one response from each NICU. There was only one case where there were three respondents for the same unit, for which one respondent was randomly selected.

Snowball sampling was used to invite participants to participate in the study. An e-mail was distributed to European members of the SCENE network asking them to answer the questionnaire and encouraging them to forward the invitation to NICUs in their country and to their European NICU contacts. The invitation e-mail specified that the questionnaire should be answered by those staff members who best knew the current feeding practices in the unit. Consent was given by the participants to complete the online questionnaire. The online questionnaire was open for answers from September 1 until November 24, 2023.

### Statistical analyses

Descriptive statistics are given as the means with standard deviations (SDs), medians with interquartile ranges (IQRs), or frequencies (percentages), according to the type and distribution of the data. Written answers to open questions are presented to illustrate current practices.

## Results

### Characteristics of participating NICUs

Fifteen countries and a total of 105 NICUs participated in the study: Belgium (*n* = 1), Denmark (*n* = 11), Finland (*n* = 2), France (*n* = 26), Germany (*n* = 1), Hungary (*n* = 3), Iceland (*n* = 2), Italy (*n* = 1), Latvia (*n* = 1), Lithuania (*n* = 2), the Netherlands (*n* = 3), Norway (*n* = 16), Sweden (*n* = 6), Ukraine (*n* = 1) and the UK (*n* = 29). The characteristics of the participating NICUs are presented in Table 1.

### Usage of skin-to-skin contact

Less than half (46%) of the NICUs had no restrictions upon placing the infant in SSC with the parents (Table 2). Apart from the reasons stated in Table 2, some units did not allow SSC when infants were less than 24–30 gestational weeks or had a birthweight lower than 500–1,000 g. Other stated exclusion criteria included treatment with cooling, critically ill infant/medically unstable, and chest drain. Approximately half (49%) of the participating NICUs stated that infants had SSC within the first hour after birth. There was a large variation between units in how much time on average the infants were assumed to spend in SSC during the first week (Table 2).

### Information provided to parents

The majority of units (91%) stated that mothers were informed about milk expression and breastfeeding before birth, if possible, or within the first six hours after birth. A majority of NICUs (88%) stated that they taught parents about sleep-awake stages, and 97% taught parents about infant feeding cues. The information on cues was mainly provided to parents verbally by the nurse allocated to the infant/parents on that day (91%), by a designated team (36%) or through written material (39%).

### Practices related to observing and responding to infant feeding cues and the assessment of milk intake

One-third of the NICUs had restrictions for initiating breastfeeding (Table 3).

Nearly half (47%) of the NICUs would not normally assess the intake of milk by breastfeeding within the first week of life for someone like Olivia. For those who would, several methods for assessing milk intake were presented (Table 4). At 33–35 gestational weeks, a large variety of methods remained, and most units used different types of “estimation” without the use of a scale (Table 4).

### Staff training, guidelines and tools

Most NICUs (80%) provided new nurses with training on feeding cues within the first six months of employment. Most units (72%) also reported that it was the nurse who was assigned to the family that day who held the power of decision making regarding the feeding. Many guidelines and tools were available for nurses. In 78% of the NICUs, there existed guidelines on SSC, initiation of breastfeeding, feeding cues, frequency/timing of feeding, and (47%) reported that tools/instruments supported the transition from tube feeding to breastfeeding. A large number of different instruments/scales were used in NICUs, such as the Neonatal Feeding Assessment Scale [35], the Preterm Infant Breastfeeding Behavior Scale [36], the Breastfeeding Wheel [27] and UNICEF Baby Friendly Initiative breastfeeding cues [4]. Additionally, several locally developed or revised scales were listed [24, 27, 35, 36].

### Support during early discharge

Most of the NICUs (83%) stated that maintaining a stable temperature and “growing well” were criteria for discharge. In half of the NICUs, the infants had to be fed exclusively orally before discharge, and nearly half of the NICUs (47%) used a certain gestational age, ranging from 33 to 37 gestational weeks, as a criterion. Other criteria stated included: at least two people at home, family happy for discharge or a certain number of oral feeds per day.

Many NICUs (65%) stated that they offered specific support to enable home tube feeding discharge before the infant was exclusively orally fed. Some units provided follow-up care in which the family was visited in their home by a team/designated nurses 2–3 times/week and discharged from “home care” when the infant no longer needed tube feeding to gain weight. Other NICUs provided support at the hospital, with parents visiting the NICU a few times per week for guidance, weight checks, and so forth. Some units used digital home support, in which parents and staff held video consultations until the infant was fed exclusively by breast or bottle. In some units this was offered as the only option, while in other units video consultations were used in combination with visits at home from staff or with family coming to the hospital.

## Discussion

This study of practices supporting cue-based breastfeeding across 105 European NICUs from 15 countries showed that most NICUs trained their nurses in assessing infants’ feeding cues, informed parents about the initiation of breastfeeding, identified infants’ sleep-awake stages and feeding cues and encouraged breastfeeding whenever the infant showed cues, regardless of scheduled tube feeding times. However, many units had restrictions as to when SSC and breastfeeding could be initiated. The findings also showed a large variation in the different methods used to assess milk intake and methods for supplemental feeding until breastfeeding was established. Furthermore, many NICUs stated that they offered feeding support to families at home before discharge.

### Implementation of immediate skin-to-skin contact

The guidelines of the World Health Organization (WHO) emphasize the numerous benefits of immediate SSC for infant-mother dyads, including thermoregulation, physiological stability, stress reduction, bonding and breastfeeding support [37]. However, our survey findings indicate that while most NICUs supported SSC within 24 h of birth, fewer than 50% adhered to the WHO’s recommendation for immediate SSC. Consistent with a previous Nordic study [37], we found that 66% of NICUs imposed restrictions as to when SSC can begin, further limiting the potential benefits for both infants and mothers.

There was also a large variation in duration, with some units not allowing daily SSC. The degree of implementation of immediate SSC was highly variable [38], despite strong recommendations for immediate SSC for all preterm infants [38–42]. Complex skills and knowledge are needed to carry out immediate SSC for the smallest preterm infants [43, 44]. However, this study confirmed slow implementation even in the group of moderately preterm infants. While immediate SSC depends on preparation and an organized NICU team, it should not be controversial in cases such as Olivia [44–46]. Not only initiation of SSC but its duration has been shown to have an impact on exclusive breastfeeding [47] and should be promoted throughout the NICU hospitalization. An expected increase in the implementation of immediate SSC in the next few years could impact early breastfeeding since SSC has been shown to play an important role in initiating and facilitating direct breastfeeding [37].

### Observing and responding to infants’ cues

Many NICUs had restrictions for initiating breastfeeding, and it remains unclear whether these restrictions stem from tradition and/or safety issues. There is clear evidence indicating that while early breastfeeding is safe and should not be restricted [5, 24, 48], it should be facilitated and adjusted for the individual infant. Strategies to support early breastfeeding could and should be provided as early as possible, including for very preterm infants.

Our findings showed that 48% of the NICUs encouraged breastfeeding whenever the infant showed cues, regardless of scheduled tube feeding times during the first week of life and increased to 59% in weeks 33–35. Thus, there seems to be increasing awareness about cue-based feeding, as shown in the literature and in our findings, but in many NICUs there are significant obstacles in the form of restrictions, rules and feeding schedules.

In a recent meta-ethnographic review [28], the most prominent facilitating factors for experiencing a cue-based breastfeeding were being in close physical proximity to the infant, receiving meaningful and sensitive staff support, experiencing positive staff-mother interpersonal relationships, and being able to breastfeed when the infant signalled. Closeness, staff support and fewer barriers, such as strict feeding schedules, might be keys to success for cue-based breastfeeding. Cue-based feeding performed by parents has been reported to contribute to earlier full oral feeding in fewer days compared to traditional scheduled feedings [49]. The scarcity of empirical evidence to advocate for strategies to support cue-based breastfeeding remains a challenge.

### Assessment of breast milk intake

Our findings showed that 48% of the NICUs used test-weighing (i.e. weighing before and after breastfeeding) as a method for measuring intake. Many NICUs also had a practice whereby breast milk intake was assessed through estimating the time spent breastfeeding or through an assessment made by the mother or a nurse. The use of test-weighing infants has been studied more than other transitional strategies. There are reports about how focusing on these quantified measurements is known to be stressful for mothers [50], while others have found that test-weighing was positive for exclusive breastfeeding at discharge in preterm infants but was not associated with earlier establishment of exclusive breastfeeding [51]. The use of test-weighing has also been shown to be useful for early discharge follow-up at home [33]. The facilitation of early breastfeeding in preterm infants constitutes the dilemma of ensuring sufficient nutrition and growth while practicing breastfeeding. A recently published study reported decreased weight gain among preterm infants when cue-based feeding was used compared to a volume fed group [52]. However, the findings showed no differences in weight at discharge, and infants in the cue-based feeding group experienced full oral feeding at an earlier gestational age [53]. Securing growth, and hence breast milk intake, is of outmost concern as poor postnatal growth is associated with fewer beneficial neurological outcomes [53–55]. How cue-based approaches impact preterm infants’ growth has not been sufficiently explored. It is reasonable to assume that the dilemma of supporting cue-based feeding, based on infant cues and sleep-awake stages, while ensuring sufficient growth, may partly explain the slow implementation of cue-based breastfeeding. Our results show that while several methods are in use today, we lack knowledge on outcomes in terms of breastfeeding progression and parental experiences. Thus, there is a need to design and evaluate potentially supportive strategies enabling cue-based breastfeeding. This approach advocates a dyadic approach that entails more than just addressing lactation, breastfeeding or nutrients; it encompasses an understanding of the multifaceted influences on breastfeeding, including infant condition *and* growth, maternal health and preferences, and staff dynamics.

### Strengths and limitations

The strength of this study lies in the substantial number of NICUs that participated and the diverse representation of countries. While other large-scale studies have provided unique insights into how breastfeeding is facilitated in NICUs [56], this is the first study to specifically examine practices supporting cue-based breastfeeding. By employing the case of Olivia in the questionnaire, we aimed to capture what is practiced in daily clinical settings, rather than merely what “should be done” according to guidelines. However, the choice of case may have resulted in the omission of nuanced aspects of practice. Furthermore, we had no control over who completed the questionnaire, nor do we know how many units received the questionnaire and had the opportunity to respond but chose not to. It is likely that units with a self-perception of having supportive practices were more inclined to respond, whereas units that did not actively work with cue-based feeding may have declined. In this paper, the focus is on cue based feeding related to breastfeeding. However, many preterm infants receive breast milk and/or formula through cup and/or bottle feeding, which was also the case in the participating units of this study. Cue-based feeding is equally important when other feeding methodes are used and should also be evaluated. Another significant limitation is that we cannot ascertain whether respondents reported their actual practices or what they believed should be done. To address this, research needs to be undertaken to gather insights from parents. This is an area that warrants further exploration in future research.

### Clinical implications

Recognizing the significance of cue-based breastfeeding aligns with emerging knowledge on the importance of supporting neurodevelopmental NICU care. Our results reveal a large diversity of methods used to assess breast milk intake, although none consider reliability or personal experiences to any great extent. The evidence to facilitate cue-based breastfeeding and to support a dyadic feeding approach between the infant-mother/father is lacking. Providing adequate support to parents requires a comprehensive understanding of feeding physiology, practical skills and emotional support. Hence, nurses in the NICU must receive sufficient education and resources to effectively support parent-infant relationships and facilitate cue-based feeding and breastfeeding. However, this is not only a nurse or lactation consultant issue. Building sufficient knowledge and evidence requires efforts from a team of multidisciplinary healthcare professionals. Fostering developmental supportive breastfeeding requires not only available resources but also shared competence and involvement among multidisciplinary healthcare professionals and parents in collaboration. The largest area for improvement is to facilitate cue-based feeding and breastfeeding, and yet be able to assess intake using methods that do not stress either mothers or infants. Most importantly, we need to evaluate which strategies/interventions work best for infants and parents.

## Conclusion

This study of practices to support cue-based breastfeeding for preterm infants in 105 European NICUs showed that most NICUs encouraged breastfeeding whenever the infant showed cues, regardless of scheduled tube feeding times. However, many units had restrictions for when SSC and breastfeeding could be initiated. Further, the challenge of supporting neurodevelopmental feeding progress based on infant cues while ensuring sufficient growth was evident in the large number of strategies used to assess breast milk intake during breastfeeding. There is an urgent need for the implementation and evaluation of potentially supportive strategies that acknowledge infants’ ability to lead the way.

**Table 1:** Characteristics of participating NICUs (*n* = 105)

		*n*	%	
**Type of NICU design**				
Only single-family rooms		17	16	
Only open bay rooms		34	32	
Combination of single-family rooms and open bay rooms		43	41	
Other types of design		11	10	
**Designated number of infants in the NICU**				
6–15		42	30	
16–25		28	27	
26–35		20	29	
> 36		15	14	
**Level of care**				
Level 1		0	0	
Level 2		26	25	
Level 3 A		26	25	
Level 3B		37	35	
Level 3 C		8	8	
Other		8	8	

Level 1 = Basic care of stable infants born at 35 to less than 37 weeks gestation. Level 2 = Specialty care of infants born at least 32 weeks gestation or 1,500 g, with possibility of brief mechanical ventilation or CPAP. Level 3 A = Subspecialty intensive care of infants born at least 28 weeks gestation or 1,000 g with possibility of mechanical ventilation. Level 3B = Subspecialty intensive care of infants born at less than 28 weeks gestation or 1,000 g, with possibility of advanced respiratory support and access to paediatric surgical specialist. Level 3 C = As level 3B but including extracorporeal membrane oxygenation and surgical repair of complex congenital cardiac malformation

**Table 2:** Practices for SSC during the first week in NICUs (*n* = 105)

		*n*	%	
**Restrictions for SSC; possible to choose more than one option**				
No restrictions		48	46	
Being on a ventilator		16	15	
Having a nasal CPAP		1	1	
Having high-flow nasal therapy/high-flow nasal cannula		1	1	
Having umbilical catheter		20	19	
**Initiation of SSC for at least 1 h; in infants born at > 32 weeks GA and with a need for CPAP**				
Immediately or within 5 min after birth		25	24	
After the first 5 min but within the first hour		26	25	
During the second to twenty-fourth hour of life		39	37	
After the first day		7	7	
Other		8	8	
**Estimated duration of SSC the first week after birth; in infants born at > 32 weeks GA and with a need for CPAP**				
< 1 h/day		2	2	
1 – < 3 h/day		31	30	
3–6 h/day		37	35	
7–11 h/day		13	12	
> 11 h/day		10	19	
Other		12	11	

CPAP = Continuous Positive Airway Pressure

**Table 3:** Restrictions for breastfeeding in NICUs (*n* = 105)

		*n*	%		
**Restrictions for initiating breastfeeding, i.e., put to the breast; possible to choose more than one option**					
No restrictions		34	32		
Being on a ventilator		51	49		
Having a nasal CPAP		26	25		
Having high-flow nasal therapy/high-flow nasal cannula		7	7		
Having umbilical catheter		18	17		

CPAP = Continuous Positive Airway Pressure

**Table 4:** Responding to feeding cues and assessment of milk intake in NICUs (*n* = 105)

		Weeks 32–33		Weeks 33–35		
		*n*	%	*n*	%	
**When the mother is present, when would the infant be put to the breast**						
The infant would not be put to the breast		3	3	1	1	
At the scheduled tube feeding times, regardless of infant’s cues		7	7	6	6	
Whenever the infant showed cues at a scheduled tube feeding time		35	33	31	31	
Whenever the infant showed cues, regardless of scheduled tube feeding time		51	48	62	59	
Other*		9	9	4	4	
*During SSC, depending on the nurse, not on CPAP, combination of the above						
**How is breast milk intake assessed; possible to choose more than one option**						
Not assessed		50	47	8	8	
Weighing before and after breastfeeding		22	21	50	48	
Monitoring by daily weight		21	20	40	38	
Estimating intake by time spent sucking		18	17	40	38	
Estimating intake by the mother’s own assessment		23	22	40	38	
Estimating intake by the nurse’s assessment		26	25	40	38	
Other*		11	11	20	18	

*Breastfeeding assessment form, assessing urine and stool output as appropriate to age, weight every second day

## Data Availability

No datasets were generated or analysed during the current study.

## Abbreviations

NICU: Neonatal intensive care unit
PMA: Postmenstrual age
SSC: Skin-to-skin contact
